# Microbial Anaerobic Digestion (Bio-Digesters) as an Approach to the Decontamination of Animal Wastes in Pollution Control and the Generation of Renewable Energy

**DOI:** 10.3390/ijerph10094390

**Published:** 2013-09-17

**Authors:** Christy E. Manyi-Loh, Sampson N. Mamphweli, Edson L. Meyer, Anthony I. Okoh, Golden Makaka, Michael Simon

**Affiliations:** 1 Fort Hare Institute of Technology, University of Fort Hare, Alice Campus, Alice 5700, Eastern Cape Province, South Africa; E-Mails: smamphweli@ufh.ac.za (S.N.M.); emeyer@ufh.ac.za (E.L.M.); msimon@ufh.ac.za (M.S.); 2 Applied and Environmental Microbiology Research Group (AEMREG), Department of Biochemistry and Microbiology, University of Fort Hare, Alice Campus, Alice 5700, Eastern Cape Province, South Africa; E-Mail: aokoh@ufh.ac.za; 3 Department of Physics, University of Fort Hare, Alice Campus, Alice 5700, Eastern Cape Province, South Africa; E-Mail: gmakaka@ufh.ac.za

**Keywords:** biomass, animal wastes, anaerobic digestion, biodigester, public health

## Abstract

With an ever increasing population rate; a vast array of biomass wastes rich in organic and inorganic nutrients as well as pathogenic microorganisms will result from the diversified human, industrial and agricultural activities. Anaerobic digestion is applauded as one of the best ways to properly handle and manage these wastes. Animal wastes have been recognized as suitable substrates for anaerobic digestion process, a natural biological process in which complex organic materials are broken down into simpler molecules in the absence of oxygen by the concerted activities of four sets of metabolically linked microorganisms. This process occurs in an airtight chamber (biodigester) via four stages represented by hydrolytic, acidogenic, acetogenic and methanogenic microorganisms. The microbial population and structure can be identified by the combined use of culture-based, microscopic and molecular techniques. Overall, the process is affected by bio-digester design, operational factors and manure characteristics. The purpose of anaerobic digestion is the production of a renewable energy source (biogas) and an odor free nutrient-rich fertilizer. Conversely, if animal wastes are accidentally found in the environment, it can cause a drastic chain of environmental and public health complications.

## 1. Introduction

Biomass encompasses materials derived from plants, animals, humans as well as their wastes. In addition, food processing, agro-industrial and industrial wastes are other sources of biomass wastes, so also is the microbial population metabolically active and cultivable plus metabolically active but non-cultivable cells existing within these wastes. Depending on the characteristics of these wastes, they can be converted into energy/and or fuel by combustion, gasification, co-firing with other fuels and ultimately by anaerobic digestion [1]. 

So far, the conventional sources of energy that have provided power for developing and maintaining the technologically advanced modern world are the fossil fuels including coal, oil and natural gas. Yet, fossil resources are finite and their continued recovery and use appreciably impact our environment and affect the global climate due to the emission of greenhouse gases. Moreover, shortening of oil and gas are becoming imminent and to prepare for a transition to more sustainable sources of energy, viable alternatives for conservation, supplementation and replacement must be explored [2]. In this regard, biomass materials have been viewed as a way to expand energy supply, help mitigate growing dependence on fossil fuels and alleviate environmental and health hazards emanating as side effects from the use of fossil resources in many developing and developed countries [3].

Anaerobic digestion of biomass wastes could have a huge impact on renewable energy requirements. It is best suited to convert organic wastes from agriculture, livestock, industries, municipalities and other human activities into energy and fertilizer. It has become popular in developing countries such as China, India and Nepal; however, in South Africa, biogas digesters are principally constructed and installed in the Western and Kwa-Zulu Natal provinces of the country [4]. Owing to the important roles demonstrated by rumen microorganisms in anaerobic digestion [3], animal manures have been established as suitable sources of biogas production in Africa although, they are co-digested with energy crops in Denmark and Germany [5,6]. Co-digestion refers to the simultaneous anaerobic digestion of multiple organic wastes in one digester. This principle enhances methane yield due to positive synergisms established in the digestion medium, bacterial diversities in different wastes and the supply of missing nutrients by the co-substrates [7]. 

Furthermore, these wastes obtained from different animals vary in chemical composition and physical forms as a result of principal differences in the digestive physiology of the various species, the composition and form of diet, the stage of growth of the animal and lastly the management system of waste collection and storage [8]. Moreover, Sakar *et al*. [9] and St-Pierre and Wright [10] stated that a large proportion of the agricultural sector in both developing and developed countries is involved with poultry and livestock farming resulting in huge quantities of animal manure and other wastes from livestock operations which merit public, environmental and social concerns. Consequently numerous digesters are designed and installed on farms for the proper management of these wastes [11]. 

Overall, anaerobic digestion reduces biomass wastes and mitigates a wide spectrum of environmental undesirables, it improves sanitation, helps in air and water pollution control and reduces greenhouse gas emissions. Also, it provides a high-quality nutrient-rich fertilizer and yield energy in the form of biogas. The uses of biogas vary greatly from developing to developed countries. In Africa, biogas generated can be used as fuel for cooking, lighting and heating; it reduces the demand for wood and charcoal for cooking therefore helps preserve forested areas and natural vegetation, and can also help alleviate a very serious health problem due to poor indoor air quality associated with wood and charcoal used for cooking [12,13]. 

In Western countries (e.g., Germany & America), biogas is converted to electricity and heat for on-farm purposes by combined heat and power units after removing water and sulphur from its mixture [14]. Alternatively, it is upgraded to bio-methane constituting 95–99% methane wherein it opens up more utilization opportunities. Bio-methane is fed into the gas grid and used as power, transportation fuel and for heating [15]. 

Against this background, this paper appraises insights on environmental and public health implications arising from improper disposal of animal wastes and a comprehensive description of anaerobic digestion of these animal wastes as a means of resolving the ills; with emphasis on types of bio-digesters, microbial communities engaged in the process and techniques for their identification as well as factors affecting the digestion process.

## 2. Environmental and Public Health Implications of Animal Manure

**Figure 1:**
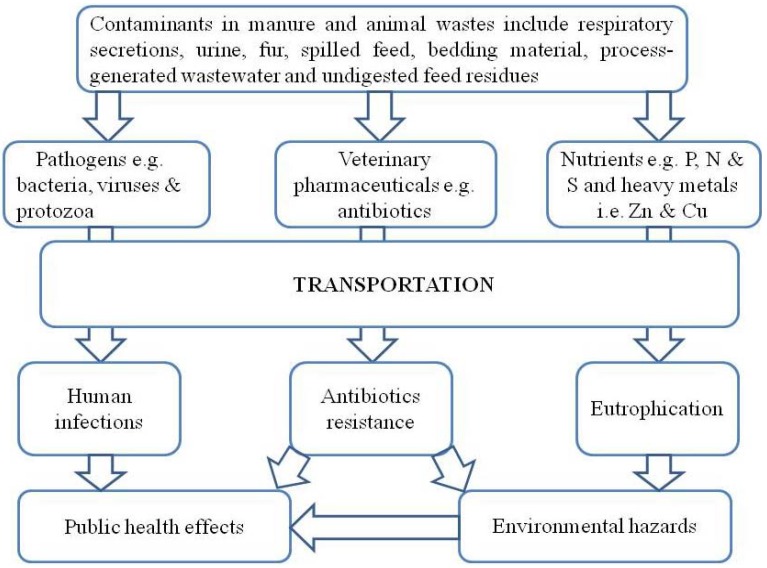
Environmental and public health implications of animal manure.

Wastes from agricultural animals (poultry and livestock) often contain high concentrations of human pathogens, spilled feed, bedding material, fur, process-generated wastewater, undigested feed residues, feces and urine therefore must be effectively managed to minimize environmental and public health risks. However, the type and pathogenic microbial load depend on the type of the waste and its composition [16]. Figure 1 shows an overview of different contaminants in animal wastes and plausible implications. The following contaminants including pathogens (bacteria, viruses and protozoa), nutrients (phosphorus, nitrogen and sulphur), heavy metals (zinc and copper), veterinary pharmaceuticals (antibiotics) and naturally excreted hormones are present in animal manure [17]. 

### 2.1. Sources of Contaminants in Animal Manure

The intestinal tract of human and animals have been found to be the major sources of *Salmonella* and *Escherichia coli* in nature [18], which could be shed in feces. These pathogens may persist for days to weeks to months depending on the type of pathogen, the medium and the environmental conditions. Approximately 1% to 3% of all domestic animals are infected with Salmonellae [12,19]. Furthermore, other non bacterial pathogens that may be present with fecal material include protozoa (*Cyptosporidium* and *Giardia*) and viruses (Swine Hepatitis E- virus). The management and disposal of animal wastes harboring such pathogens can increase the risk of infections and diseases that threatens human health if these wastes are not properly treated and contained [20].

Antibiotics are routinely used in animal farming to prevent the spread of diseases or treat infected animals and simply added to animal feed to promote/increase growth. Further compounding the problem is the fact that the misuse or overuse of antibiotics could speed up the development of resistance or increase resistance of the microbial population present [17] due to the fact that resistance genes may be transferred between the microbial communities present. The use of any one antibiotic can yield resistance to multiple antibiotics. However, it is devastating that most antibiotics are designed to be quickly excreted from the treated organisms. Thus, they are commonly found in animal wastes. 

Consequently, microorganisms in animal manures are thought to affect human health via multiple pathways either through direct or indirect contact with food, water, air or anywhere manure goes [21]. Further transmission of pathogens off-farm from farm workers to family members is also possible. It has been noted that most human-acquired infections result from these resistant strains [22]. Antimicrobial resistance of microorganisms is a local, national and global challenge; therefore the quest to identify the sources of these antibiotic resistant microbes and to seek for means to stop the spread of their resistance genes or not to select for further resistant strains is imperative.

Furthermore, animal manure may cause environmental pollution of water bodies as it is described to be rich in nutrients. Seppälä *et al*. [23] noted that these wastes harbor both micro and macronutrients including zinc and copper. These metals (Zn & Cu) are micronutrients found in animal manure originating from feed, supplements, antibiotics and water consumed by the animals [9].

### 2.2. Adverse Effects of Animal Wastes on the Environment and Humans

Taking into consideration the concentration of contaminants in animal wastes, it does have the potential to pollute land, water and air if containment and treatment do not adequately manage it. Haulage of these contaminants in animal wastes is dependent on the chemical characteristics, soil properties, climatic conditions and crop management practices. It is most probable that rain may wash/flush these wastes into streams, rivers or may cause waste to seep through the soil into underground springs and wells that humans use for sanitation and domestic purposes [24]. 

From the environmental point of view, excessive nutrients (especially phosphorus and nitrogen) in conjunction with elevated levels of biological oxygen demand (BOD) and chemical oxygen demand (COD) in these water bodies can contribute to algal blooms and cyanobacterial growth thus presenting serious socioeconomic hazards [25]. As a long term effect, they may cause shifts in phytoplankton community structure from desirable species to noxious species by means of holding back the growth of desirable aquatic species. Antibiotics in the soil may affect the natural ecosystem functions such as soil microbial activity and bacterial denitrification [17].

Of profound public health effect is the reality that waterborne pathogens can be accidentally ingested during recreational activities or drinking water that is contaminated with animal feces [26]. This might result in acute gastrointestinal upset, e.g., nausea, diarrhea and vomiting. Also, contact with affected surface water during recreational activities can cause skin, ear or eye infection. Cyanobacteria (blue green algae) in surface water can produce neuro-toxins and hepatotoxins which are known to cause acute and chronic health complications [27]. However, the disease conditions in susceptible individuals including the very young, the elderly, pregnant women, and immunocompromised may be more severe, chronic or at times fatal [26]. In addition, antibiotic resistance interferes with antimicrobial chemotherapy causing treatment failure of some infections thereby presenting life threatening situations. Consequently, anaerobic digestion of animal manure in bio-digesters can substantially help to address the above-mentioned troubles arising from the improper or careless disposal of these wastes.

## 3. Anaerobic Digestion of Animal Wastes in Bio-Digesters

Growth and intensification of livestock operations often result to great quantities of manure that have to be properly managed. Even when stored, manure generates and releases methane (a greenhouse gas) into the atmosphere [10]. Moreover, anaerobic degradation has usually taken place in the lower digestive tract of animals and then continues in the manure piles resulting in malodorous compounds. These malodorous compounds originate from the incomplete breakdown of organic matter in manure by anaerobic microbes under uncontrolled environmental conditions [28]. 

Farm-based anaerobic digester presents as an alternative to the proper management of these wastes [29]. There are thousands of on-farm digester plants worldwide including Blue Spruce Farm, Green Mountain Diary, Chaput Family Farm, Cantabria Diary Plant, Buttermilk Hall Farm, Bulcote Farm, Minnesota mid-sized Diary Farm and a host of others [10,30]. However, Lutge and Standish [31] noted that very few on-farm anaerobic digesters are available in South Africa. Nevertheless, the type of digester used is being influenced by the characteristics of manure collected which is dependent on the animal’s diet and on-farm practices [32]. All the same, these farms employ anaerobic digestion which is a consistent technology for their waste management/treatment.

Taking into consideration the aforementioned differences in livestock practices existing between the farms, the essential step of liquid-solid separation of manure mixture may be performed before or after anaerobic treatment [33]. In addition, the manure collected may be mixed with milk house waste for anaerobic digestion [34]. Generally, manure is collected with or without milk house waste and slurry prepared by adding water to it. The slurry is pumped to the separator for screening, separating the mixture into liquid and solid fractions. Subsequently, the screened liquid fraction is fed into the digester whilst the solid fraction could be dewatered and redistributed to areas lacking nutrients, used as bedding and or composted to serve as an additional source of more carbon and nitrogen [9]. In addition, the digested liquid fraction can be processed to obtain concentrated fertilizers or post-treated to obtain clean water for recycling and irrigation purposes [30,32]. 

**Figure 2:**
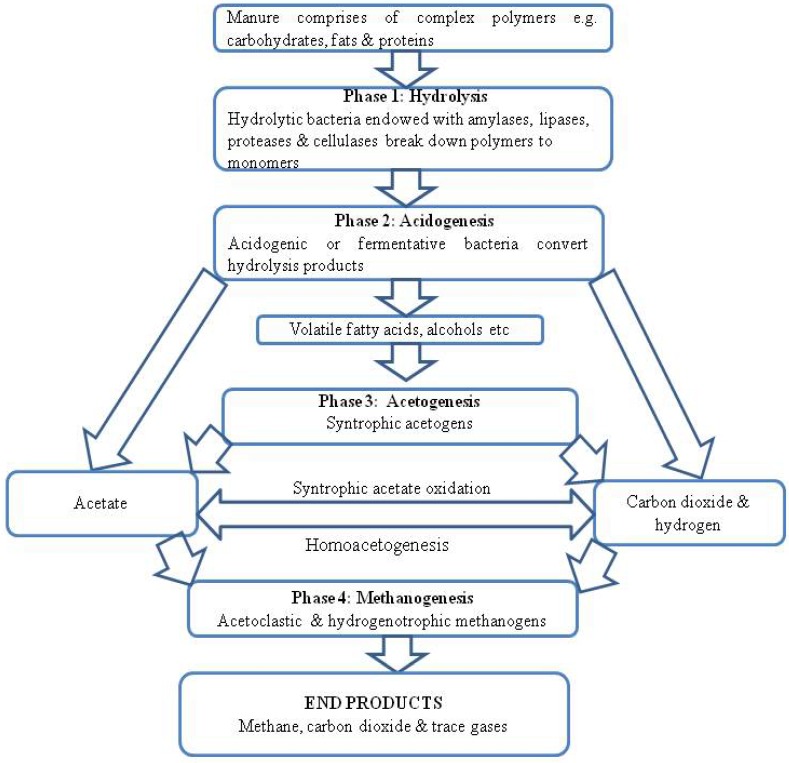
A schematic presentation of anaerobic digestion process.

Overall, during anaerobic digestion complex polymers in animal wastes are catabolized through a series of steps by complex consortia of microorganisms in the digester to ultimately yield methane and carbon dioxide [35]. Basically, this process can be divided into four phases: hydrolysis, acidogenesis, acetogenesis and methanogenesis in which hydrolytic, fermentative bacteria, acetogens and methanogens play distinct roles, respectively, as shown in Figure 2. 

During hydrolysis, complex polymers like carbohydrates, proteins and fats are being degraded into sugars, amino acids and long chain fatty acids respectively. This breaking down process occurs primarily through the activity of extracellular enzymes (lipases, proteases, cellulases & amylases) secreted by hydrolytic bacteria attached to a polymeric substrate [36]. 

Later, fermentative or acidogenic bacteria transform hydrolysis products into acetic acid and intermediate compounds, such as ethanol, lactic acid, short chain fatty acids (C3–C6), hydrogen and carbon dioxide [35]. Acetate, carbon dioxide, formate, methylamines, methyl sulphide, acetone and methanol produced in this phase can be directly utilized for methanogenesis. Consequently, the other intermediary products from acidogenesis are converted to acetate, formate or CO_2_ & H_2_ by syntrophic acetogens in a bid to maximize methane production. 

As a final point, methane is produced during methanogenesis by methanogens in two ways: either through cleavage of acetic acid molecules to produce methane and carbon dioxide or reduction of carbon dioxide with hydrogen by acetotrophic and hydrogenotrophic methanogens, respectively [37]. The biogas generated constitutes mainly methane (50–75%), CO_2_ (25–45%) and traces of other gases like CO, H_2_S, NH_3_, O_2_, water vapor (Table 1). 

**Table 1:** Various constituents of biogas generated from the anaerobic digestion process; its average composition adopted from de Graaf and Fendler, [38].

Component	Symbol	Percentage
Methane	CH_4_	50–75
Carbon dioxide	CO_2_	25–45
Hydrogen	H_2_	1–2
Ammonia	NH_3_	<1
Water vapor	H_2_O	2–7
Oxygen	O_2_	<2
Hydrogen sulphide	H_2_S	<1

### 3.1. Microbial Communities Involved in Anaerobic Digestion of Animal Manure and Methods of Their Identification

The specific microbes and their metabolic activities during anaerobic digestion depend on the chemical composition of the feedstock/waste, environmental factors and digester operating conditions [39,40]. Four sets of microorganisms are involved and these groups of microorganisms are tightly attached metabolically whereby the early stages of digestion yield reduced intermediate products that are utilized by acetogens and methanogens [37]. However, the interrelationship between the acetogens and methanogens is highly complex. These microorganisms are classified as anaerobes therefore oxygen poses a threat via the disruption of metabolic pathways causing oxidation of cellular factors that normally occur in reduced form. Contrarily, in recent times, it has been documented that several methanogens adapt to oxygen due to the presence of genes that synthesize enzymes (e.g., catalase and superoxide dismutase) within their genomes, which serve a role in defense against oxygen toxicity [41]. Several authors have reported the high tolerance of methanogens including *Methanobacterium thermoautotrophicum*, *Methanobrevibacter arboriphilus* and *Methanosarcina barkerii* to oxygen and dessication [42,43]. 

More elaborately, Anderson *et al*. [44] also noted that after dessication process, *M. barkeri* had innate capability to survive extended periods of exposure to air and lethal temperatures owing to the synthesis of thick outer cell layers composed of extracellular polysaccharide (EPS); added to the accumulation of cyclic 2,3-diphosphoglycerate (a novel metabolite which may be used to stabilize proteins at elevated temperatures). In addition, the membrane lipids of archael species have glycerol molecules bound by ether linkages to branched isoprene hydrocarbon molecules causing the organisms to adjust to such extreme temperatures [45]. On the whole, microbial community within a digester system can be grouped into acidogens, syntrophic acetogens and methanogens [46].

#### 3.1.1. Acidogens

It has been documented that the bacterial species active in the polymer hydrolysis phase are also active during the acidogenic phase. Hence, the hydrolytic and acidogenic bacteria are sometimes referred to as fermentative bacteria. They can be either facultative anaerobic bacteria (*i.e.*, can survive under both aerobic and anaerobic conditions) or strict anaerobes. The family *Enterobacteriaceae* or enteric bacteria (a group of bacteria that inhabit the intestine of humans and other animals) are active fermenters and are among the organisms responsible for the first step in the bioconversion of carbohydrates to CH_4_ [18]. 

In addition, Blumer-Schuette *et al*. [47] and Wirth *et al*. [48] documented the relevance to biomass deconstruction of the following microorganisms: *Caldicellulosiruptor saccharolyticus*, *Thermotoga maritima*, *Clostridium thermocellum*, *Anaerocellum thermophilum*, *Escherichia coli*, *Clostridium kluyveri*, *Bacillus cereus*, *Ruminococcus albus etc*. Other known groups of anaerobic cellulose-degrading bacteria are found in the following genera; *Aminobacterium*, *Psychrobacter*, *Anaerococcus*, *Bacteroides*, *Acetivibrio*, *Butyrivibrio*, *Halocella*, *Spirochaeta*, *Caldicellulosiruptor* and *Cellulomonas* (a facultative anaerobe of the phylum *Actinobacteria*) [49,50].

#### 3.1.2. Syntrophic Acetogens

Syntrophic acetogens, e.g., *Syntrophobacter wolinii*, *Syntrophomonas wolfei* and *Smithella sp.* are responsible for the syntrophic metabolism of alcohols, short chain fatty acids (C3–C6), some amino acids and aromatic compounds to yield methanogenesis substrates [51]. The conversion of the above-mentioned substrates to yield methanogenesis products is thermodynamically unfavorable but it becomes favorable with the presence of a syntrophic partner (hydrogenotrohphs) [51,52].

However, accumulation of volatile fatty acids results in decrease in pH, increase acidification, destroy methanogens activity and leads to failure of digester ultimately [7]. While the syntrophic acetogens are converting intermediary metabolites to acetate and other methanogenesis substrates, homoacetogens also produce acetate from the reduction of carbon dioxide with hydrogen via the acetyl Co-A pathway [53]. 

Overall, methane is produced during methanogenesis by acetotrophic, hydrogenotrophic and methylotrophic pathways. In regards to acetate degradation to yield methane via the acetoclastic pathway, specific methanogens from the order *Methanosarcinales* are responsible. Contrarily, a group of acetate-oxidizing bacteria occur in syntrophic relationships with hydrogenotrophic methanogens wherein they oxidize acetate to form methane in association with the latter microorganisms. 

These bacteria are equally referred to as syntrophic acetogens and include both mesophilic and thermophilic bacteria (Table 2). Syntrophic acetate-oxidizing bacteria are involved in the reversed reductive acetogenesis [54] and can be identified by the combinatorial use of flux measurement and transcriptional profiling of formyltetrahydrofolate synthetase (FTHFS) gene, an ecological biomarker engaged in reductive acetogenesis [52]. 

**Table 2:** Syntrophic acetate-oxidizing bacteria in association with hydrogenotrophic methanogens.

Acetate-oxidizing bacteria	Microbial description	Hydrogenotrophic methanogens	References
AOR	Anaerobic, rod-shaped, gram positive, non-spore forming and thermophilic (60 °C)	*Methanobacterium sp*. strain THF	Lee and Zinder [55]
*Clostridium ultunense*	Anaerobic, spore-forming, rod-shaped, gram negative and mesophilic (37 °C)	*Methanoculleus sp*. strain MAB1	Schnürer *et al*. [56]
*Thermacetogenium phaeum*	Anaerobic, rod-shaped, gram negative but with gram positive cell wall structure and thermophilic (between 55 and 58 °C)	*Methanothermobacter thermoautotrophicus* TM	Hattori *et al*. [57]
*Thermotoga lettingae*	Anaerobic, rod-shaped, non-spore forming, mobile, gram negative and thermophilic (65 °C)	*Methanothermobacter thermoautotrophicus* or *Thermodesulfovibrio yellowstonii*	Balk *et al*. [58]
*Syntrophaceticus schinkii*	Anaerobic, spore-forming, variable cell shape, gram variable and mesophilic (between 25 and 40 °C)	*Methanoculleus sp*. strain MAB1	Westerholm *et al*. [59]

#### 3.1.3. Methanogens (Archaea)

Methanogens are found in a wide range of anaerobic habitats including freshwater and marine habitat, sewage digesters, the digestive tracts of herbivores, mammals and wood and humus feeding insects *etc*. [60,61,62,63]. They belong to the domain *Archaea* and they occupy a key position in the anaerobic digestion process because it is in this last step of the process where the valuable methane is produced [45]. During an unstable anaerobic digestion process in a poorly performing anaerobic digester, the methanogenic populations are especially sensitive to acidity (pH), concentrations of volatile fatty acids, and free ammonia and ammonium ions in the digesting substrate [64].

In addition, methanogens are classified into six orders *i.e.*, *Methanobacteriales*, *Methanococcales*, *Methanomicrobiales*, *Methanosarcinales*, *Methanocellales* and *Methanopyrales* [65]. Members of the order *Methanosarcinales* utilize acetate which has for a long time been known as the major precursor for more than 70% of methane produced in most engineered anaerobic digester [66]. In other words, previous knowledge assumed that in totality, two-thirds of methane are obtained from the acetoclastic methanogenesis pathway and one-third from hydrogenotrophic methanogenesis pathway according to anaerobic digestion model 1 (ADM1) [66]. This is misleading since reports were based on anaerobic digestion of waste water and sewage sludge with very low organic content. 

Clearly, biomethanation involves a complex community of specialized microorganisms that depend on each other either for substrate supply or metabolism of end products in order to favor their metabolic activity. Moreover, microbial species require specific combination of physical and chemical conditions viz temperature, pH, salinity besides substrate availability to thrive. Therefore, microbial species obtained from different environment associated with different physical and chemical factors tend to vary even though they could perform anaerobic digestion through the same stages of hydrolysis, acidogenesis, acetogenesis and methanogenesis [10]. However, this might influence the biogas yield obtained under different physical, chemical and substrate conditions.

Nowadays, results obtained from several studies conducted by different authors have contradicted the assumptions of ADM1. Krakat *et al*. [67,68] demonstrated that a higher temperature of 60 °C and a drastic reduction in hydraulic retention time resulted in dominance of hydrogenotrophs among the microbial communities with a corresponding increase in methane production in thermophilic and mesophilic biogas fermentors respectively, digesting energy crops. Similarly, Klocke *et al*. [69,70] reported varying substrate utilization during methanogenesis within the biogas plant as depicted from the dominance fraction of hydrogenotrophic methanogens relative to acetoclastic counterparts.

Furthermore, members of *Methanosarcinales* are described as acetoclastic and comprise of two families *Methanosarcinaceae* and *Methanosaetaceae*. However, these two families of acetoclastic methanogens differ in their physiology, biokinetics and growth environment with respect to the acetate concentration [2]. Conclusively, the interactions of the different groups of anaerobic microorganisms are incredibly complicated, and the effective performance of the biological process strongly depends on the balance of these relationships [71]. 

### 3.2. Techniques for Identifying Microorganisms Involved in the Anaerobic Digestion Process

St-Pierre and Wright [10] mentioned that the microbial communities within an anaerobic digester treating animal manure are not fully characterized. However, due to the diversity of microorganisms in the system, a variety of methodological approaches are required for a detailed analysis of community structure in a bid to unravel the complex antagonistic and synergistic effects between microbial communities in order to eventually improve process stability and efficiency of biogas formation [72,73]. This can be achieved by the combined use of traditional culture-based, microscopic and molecular techniques. 

Conversely, culture-based techniques such as plate counts, membrane filtration and most probable number (MPN) have an inherent limitation because only the viable population will grow to produce colonies under specific growth conditions whereas others that are important in the original sample do not proliferate [74]. However, these traditional culturing methods employed with environmental samples also underestimate the total number of microorganisms due to the selective nature of the media used, the lack of detection of active but non cultivable (ABNC) microbes and failure to count microbes that are present as aggregates or associated with particles. Likewise, it is impossible to obtain pure cultures of most microorganisms in natural environment due to the complex syntrophic and symbiotic relationships that are abundant in nature [75]. 

Contrarily, direct microscopic methods e.g., DAPI epifluorescence microscopy allows the direct observation and total enumeration of viable and non-viable microorganisms in the feedstock [72]. Specifically, the identification and enumeration of methanogenic microbes can be achieved by epi-fluorescence microscopy, a technique based on their unique fluorescent pigment, factor F_420_ [76]. The coenzyme F_420_ shows autofluorescence (blue-green color) of methanogenic cells when excited by UV light. Hence, this autofluorescence serves as a diagnostic tool used to count autofluorescent methanogens [77]. Results from this technique can be combined with those from molecular methods for a better overview of microbial population and structure in an anaerobic digester [78].

Molecular techniques targeting particularly 16S rRNA genes (the only RNA component of 30S ribosomal subunit), can also be employed as conventional methods for identification of microbial community in a digester. These methods include cloning, fluorescent *in situ* hybridization (FISH), denaturing gradient gel electrophoresis (DGGE), single strand conformation polymorphism (SSCP), restriction fragment length polymorphism (RFLP), quantitative real-time PCR (qPCR) and DNA sequencing (Sanger and next generation sequencing methods). Each of these methods has its advantages and shortcomings that have been deliberated and presented elsewhere [74,79]. 

Most previous studies on anaerobic digestion of biomass materials were based on the construction of 16S rRNA clone libraries and subsequent sequencing (Sanger method) of individual clones [69]. The resulting sequences corresponded to different taxa that were employed in the phylogenetic classification thereby revealing the structure of the underlying community. In addition to knowledge on phylogenetic diversity, the microbial population present in an anaerobic digester can be quantified using FISH and qRT-PCR as well as uncultured microbes will be uncovered by the use of molecular methods based on 16S rRNA [80]. However, the analysis of 16S rRNA gene does not cover the whole complexity of the environment due to sequencing of limited number of clones as well as low cloning efficiencies [81].

In addition to the methods relying on 16S rRNA for the detection of microorganisms in an anaerobic digester, other methods are available incorporating other remarkable genes which could serve as a diagnostic tool. The hydrazine oxidoreductase genes (*hzo* gene) can be employed in phylogenetic diversity and functional analysis of anaerobic ammonium-oxidizing bacterium (anammox) in a community or an environment. The *hzo* genes are specific for the identification of anammox bacteria and can be used as a functional marker to give activity-based information regarding the anammox bacteria in a community [82]. Ozgun *et al*. [83] identified and quantified anammox bacteria from waste water treatment plant by adopting a FISH-based approach with the PCR primers designed for the amplification of *hzo* specific genes. 

Furthermore, hydrazine synthase (*hzs A*) also represents a unique phylogenetic marker for anammox bacteria hence; it can be used for identification purpose. Moreover, Harhangi *et al*. [84] determined the presence and biodiversity of anammox bacteria from samples of wastewater treatment systems, fresh water and marine environments as well as anammox enriched cultures by the development of PCR primer set targeting a subunit of hydrazine synthase. The tested primers successfully retrieved *hzs A* gene sequences covering all known anammox genera thus indicating that the use of 16S rRNA gene does not directly relate to the physiology of the targeted microbes and also the primer set currently available does not give tangible information pertaining to the diversity of the anammox species. 

Also, methanogens can be distinguished from other microbes by virtue of their cell wall components, unique membrane lipids, 16S rRNA gene sequences as well as key enzymes that are involved in methanogenesis. The key enzymes encompass specific coenzyme and cofactors such as F_420_, methanopterin and coenzyme M that are engage in methanogenesis [85]. These enzymes are strongly conserved and found only among methanogens. Consequently, methanogens can be specifically identified by targeting genes that code these peculiar enzymes.

Of great significance is the methyl Coenzyme M reductase (an enzyme complex) that catalyzes the reduction of the methyl group bound to coenzyme M and is encoded by the gene *mcr A*. It has been documented that methanogenic biodiversity displayed upon utilization of *mcr A* is similar to that revealed by 16S rRNA therefore validating the application of the former gene for identification [86,87]. However, a prominent drawback linked to the incorporation of *mcr A* gene is that its available sequences are very limited in the database therefore primers designed based on these sequences may lead to inefficient amplification and an adequate representation of the methanogenic community will not be delineated [85]. 

Interestingly, next generation sequencing methods (NGS) are emerging as a robust technology that creates abundant data and has caused a fundamental shift in molecular biology. These new methods involve whole genome sequencing; function-driven metagenomics and high throughput sequencing [85] that have the potential to demonstrate new insight into the entire genome of the microbial environment resulting in the rapid characterization of targeted sequences at less cost compared to the first-generation sequencing method, “traditional Sanger method” [88].

Sequencing of the whole genome is often aimed at obtaining information about the complete set of genes in any particular genome [89]. Genome sequencing opens up unexpected opportunities with information that cannot be obtained with conventional microbiological methods. For instance, Leahy and colleagues [90] unravelled the presence of vaccine and chemogenomic targets in their attempt to assess the sequencing of the M1 genome (first ever identified genome of rumen archael species) of *M. ruminatium.* Attwood *et al*. [61] equally noted the presence of a phage sequence residing within the genome of *M. ruminatium* and postulated that enzymes from the phage could serve as potential methanogen control agents. However, knowledge of the complete genetic makeup of an organism is not sufficient as information pertaining to their function is missing thus indicating that the biochemical function of each gene product is crucial. 

On the other hand, metagenomics or community genomics or environmental genomics is the analysis of genomic DNA of the whole microbial communities directly in their natural environments by extracting or isolating the total DNA from an environmental sample [91]. Specifically, function-driven metagenomics is a powerful tool that has the potential to identify entire new classes of genes of new or known functions thus providing information on the metabolic activities of all members of a microbial community, including even those that were uncultivable and undefined previously [91].

In addition, Gilbert and Dupont [92] further described that metagenomics embodies two aspects; the environmental single-gene surveys in which single targets are amplified and the PCR- amplified products are sequenced and secondly, random shotgun studies of all environmental genes where the total DNA is isolated and sequenced; giving a profile of all the genes occurring in the community. However, the extent of community coverage depends on the depth of sequencing.

With the advent of high throughput sequencing technologies such as Roche 454, Illumina/Solexa, Applied Biosystems/SOLiD and Helicos BioSciences, more sequence data are obtained than has ever been possible with the traditional Sanger sequencing method [10]. These improved sequencing methodologies do not require cloning of the DNA before sequencing thereby; they bypass one of the main biases in environmental sampling. Consequently, bioinformatics strategies and tools can be used for the analysis of the huge data obtained [85]. 

In conclusion, conventional molecular tools based on 16S rRNA (viz PCR, qRT-PCR, FISH, DGGE, cloning and sequencing of gene library) are still of relevance since they provide an initial selection of collected samples prior to comprehensive analyses by next generation sequencing technologies [85]. Moreover, from clone libraries of 16S rRNA, usually precise sequences are assigned as a result of larger sequence length relative to the short read lengths obtained from high throughput sequencing technologies [50]. Therefore, it is very promising integrating conventional molecular methods, epi-fluorescence microscopy and high throughput sequencing technologies in describing the microbial structure of a biogas plant. Combining these methods will reveal the entire complexity of the microbial communities in an anaerobic digester as well as the physiology and function/activity of the community. Also, previously unidentified microbes with culture-based techniques will be delineated and their function within the community will be recognized [91]. 

### 3.3. Types of Bio-Digesters for Treating Animal Manure

A biogas digester consists of one or more airtight reservoirs (chambers) into which animal manure or a mixture of manure and co-substrate is placed, either in batches or by continuous feed [93]. These biogas generating systems could be categorized on the basis of the number of reactors used into single (one) stage or multi (two) stage and on the mode of feeding into continuous and batch feeding systems [12]. 

In single stage processes, the three stages of anaerobic process occur in one reactor; however, the growth rate of fermentative bacteria is faster than that of acetogenic and methanogenic bacteria [33]. Consequently, acids accumulate; the pH falls and the growth of methanogenic bacteria is inhibited due to increase organic loading rate and inappropriate other process parameters. Whereas multi-stage processes make use of two or more reactors that separate the acetogenesis and methanogenesis stages in space and allows the establishment of operational conditions that reduce the start time and microbiota specialization in each reactor, thereby allowing the most desirable products at each stage to be obtained [35,69].

In a batch experimental set up, the digester is loaded with the feedstock at the beginning of the reaction and the product discharged at the end of each cycle whereas in continuous feeding, the organic material is continuously charged and discharged [12]. 

Livestock operational practices differ between individuals and they influence the characteristics of the manure obtained which in turn determines the choice of the digester [28]. Manure can be collected either by scraping with an automated device or flushing with water [32]. Ideally, scraped manure can be digested by a complete mix digester (e.g., continuously stirred tank reactor, CSTR) and a plug flow digester whereas flushed manure warrants the use of covered lagoons and anaerobic fixed film digesters [31,94]. 

Traditionally, animal manure is often flushed, pretreated by means of mechanical screening, sedimentation or both in a bid to achieve two separate fractions of liquid and sludge; with the liquid portion pushed into covered lagoons for storage and anaerobic treatment [28]. However, the anaerobic digestion process in the lagoon is affected by climatic conditions (temperature) as well as the water table on the site especially since liquid can seep into underground spring and streams [95]. In recent times, with the quest for retaining active microbial population in the digester in order to improve process stability and control; biodigesters are designed with active microbial populations attached to inert supports as biofilms or form aggregates or granules [9,94]. Sakar *et al*. [9] revealed that up flow anaerobic sludge blanket is most suitable for the anaerobic digestion of poultry wastes.

More elaborately, conventional digesters used for anaerobic treatment of animal manure are CSTR and plug-flow reactors with appreciable holding capacity though with long HRT compared to fixed film digesters [11]. On the other hand, anaerobic fixed film reactors have the potential of retaining microbial mass (as biofilms) on support materials and also reduce the retention time for anaerobic digestion to several hours or a few days [94]. However, Lutge and Standish [31] mentioned that South Africa has the potential of implementing the use of CSTR and covered lagoons for on-site animal manure treatment.

### 3.4. Factors Influencing Anaerobic Digestion of Animal Manure

Generally, factors affecting the performance of an anaerobic digester include operational factors (pH, temperature, organic loading rate (OLR)/hydraulic retention time (HRT), free ammonia concentration), substrate characteristics/biodegradability and biodigester design [96]. However, Wilkie [28] reported that temperature, biodegradability, OLR and HRT have great impact on the anaerobic digestion of animal manure. Notwithstanding, other factors should not be overlooked.

#### 3.4.1. Temperature

Based on temperature, anaerobic microorganisms can be categorized into psychrophiles (<20 °C), mesophiles (25–37 °C) and thermophiles (55–65 °C) [97]. Some methanogenic species exhibit a preference of extreme heat (90–100 °C) thus, are classified as hyperthermophilic methanogens [45]. Examples are *Methanocaldococcus jannaschii* and *Methanococcus vulcanius* [62]. Temperature can be considered as the most important environmental factor influencing the growth of microbes. Albeit, each microorganism has a certain temperature range within which it can grow and multiply. When temperature is increased within a certain range, the chemical and enzymatic reactions increase at a faster rate and growth increases [98]. 

However, above optimum temperature, key chemical reactions in the different metabolic pathways being catalyzed by enzymes cannot occur because enzymes are irreversibly destroyed since they are protein like in nature. Enzymes are crucial to metabolism because they allow organisms to drive desirable reactions that require energy and will not occur by themselves, by coupling them to spontaneous reactions that release energy. Consequently, the growth rate of microbes will equally stop [99]. However, different microbial species respond differently to abrupt changes in temperature. Moreover, temperature does not only influence the rate of metabolism of the microorganisms but also affect other process parameters such as OLR and ammonia concentration [29,100].

Generally, anaerobic digestion of biomass wastes could occur both at mesophilic (25–37 °C) and thermopilic (55–65 °C) temperature ranges. However, the ratio of free ammonia to total ammonium ion is higher at thermophilic temperature ranges. Consequently, animal wastes (containing nitrogen and ammonia compounds) are digested at mesophilic temperature range (25–37 °C) in a bid to avoid ammonia mediated inhibition of methanogenesis [29]. Moreover, thermophilic treatment requires high energy thereby may reduce the net energy obtained from the overall digestion process [100]. In spite of the abovementioned drawbacks of thermophilic fermentation; it undoubtedly causes significant destruction of pathogens and weed seeds and also causes higher metabolic rate resulting in higher methane yield [100,101].

#### 3.4.2. pH and Alkalinity

In regards to anaerobic digestion, it is more appropriate to discuss pH alongside alkalinity since the latter can be used to control pH thus buffering the acidity of the system derived from acidogenesis phase [102]. Therefore, the amount of alkalinity present in an anaerobic digester represents the buffering capacity.

The pH range of anaerobic digestion normally occurs near neutral pH range and it is dependent on the OLR (which depends on reactor type) and the buffering capacity of the substrate. Livestock wastes (rich in ammonia and nitrogen compounds) such as cow, swine and poultry manure have high buffering capacity as they produce alkalinity when degraded upon by microorganisms [103]. However, anaerobic digestion of these wastes is often maintained at higher pH values of 7.6 [29]. An increase in OLR with a corresponding decrease in HRT can result to accumulation of volatile fatty acids which causes a drop in pH due to increased acidity of the digesting medium [104]. However, in instances where the pH has to be adjusted, several chemicals such as sodium hydroxide, potassium hydrogen carbonate, sodium carbonate, calcium carbonate, calcium hydroxide *etc*. can be added for alkalinity supplementation [40]. 

#### 3.4.3. Ammonia Concentration

Anaerobic digestion of urea- and protein-rich wastes such as animal wastes is often faced with the challenge of high levels of free ammonia due to their high organic nitrogen concentration which upon biological degradation results in high concentration of total ammonium ion plus free ammonia [105]. The quantity of ammonia produced during the digestion process is attributed to substrate concentration of nitrogen, reactor loading, C/N ratio, buffering capacity and temperature. In aqueous solution, inorganic ammonia nitrogen exists in two principal forms; ammonium ion (NH_4_^+^) and unionized ammonia or free ammonia (NH_3_) in a pH dependent equilibrium state. Ammonia toxicity is influenced by the operating pH and temperature [29]. 

An increase in pH will cause increase ammonia toxicity of the system since a greater part of the total ammonia nitrogen will be free ammonia, the form that has been recognized as a toxic agent [106]. On the other hand, reduction in pH to a level within the optimum pH necessary for growth of the microorganisms will help to counteract free ammonia concentration. However, process instability provoked by ammonia toxicity often results in increased level of volatile fatty acids with a corresponding decrease in methane yield [64].

In addition, Strik *et al*. [107] noted that high ammonia concentration led to poor biogas quality requiring treatment, decreased COD removal efficiency, decreased biogas generation and malodor, besides process inhibition. High free ammonia content has usually been associated with unstable process performance and increased risk of process failure as a result of its inhibitory effect on methanogens (specifically acetate-utilizing methanogens). Therefore, in the presence of elevated levels of ammonia in a fermentor, a shift occurs in the biomethanation process from acetoclastic methanogenesis (performed by acetate-utilizing methanogens) to syntrophic acetate oxidation conducted by syntrophic acetogens in collaboration with hydrogenotrophs [64]. Moreover, El-Mashad *et al*. [100] revealed that ammonia toxicity does not only affect the acetoclastic methanogens but also hydrolysis and acidification processes.

Furthermore, chemical equilibriums especially of free ammonia concentration at a fixed total ammonium concentration can be affected by the operating temperature (*i.e.*, mesophilic and thermophilic temperature ranges) of the digester system. Even though the temperature is pivotal in the thermodynamics and kinetics of microbial reactions in methanogenesis, challenges are encountered during treatment at thermophilic temperatures (*i.e.*, 55–65 °C) of ammonium-, urea- and protein-rich biomass materials owing to a high level of free ammonia [108]. Garcia *et al*. [29] noted that at higher temperatures the ratio of free ammonia to total ammonium was much higher; consequently affecting methane generation due to free ammonia inhibition. Nevertheless, an increase in temperature within the mesophilic range relieved the digester system of ammonia toxicity. Therefore, anaerobic digestion of animal manure at mesophilic temperature offers better process stability and performance of the digester system than at thermophilic temperatures [101]. 

It has been observed that co-digesting animal wastes with carbon-rich co-substrates will help to prevent both volatile fatty acid and ammonia mediated inhibition [103]. However, the inhibitory ammonia threshold concentration is not standardized because of the conflicting results obtained from different studies conducted under different environmental conditions with different substrates and inocula in conjunction with the complex nature of the anaerobic digestion process and acclimation periods [106]. The way microbes tend to adapt to increased ammonia level is dependent on the rate of ammonia formation which is influenced by the OLR and HRT.

#### 3.4.4. Hydraulic Retention Time and Organic Loading Rate

HRT is the average period of time that the substrate resides in the anaerobic digester and OLR describes the amount of organic matter expressed in g COD/L or g TS/L or g VS/L added to the digester per reactor volume and unit time. HRT is inversely proportional to OLR and both are very useful parameters that contribute knowledge on design and performance of the reactor [109].

Biological decomposition of animal manure is affected greatly by its retention time in the reactor [110]. Retention time is determined by solid content of manure, temperature as well as the type of reactor used for treatment [11]. More elaborately, CSTR and plug flow reactors for animal manure treatment require retention time of 20–30 days whereas fixed film reactors usually have a shorter retention time of several hours to a few days [101]. However, covered lagoons require a longer retention time of 60 days [28]. In addition, HRT also affects the quality of effluent in terms of microbial load, nutrient content as well as the methane yield. Umaňa *et al*. [94] investigated the influence of HRT on anaerobic fixed bed reactor and noted that the quality of effluent and methane yield increased due to increase in HRT.

On the other hand, OLR is dependent on temperature and HRT. An abrupt increase in OLR causes system failure attributed to decreased COD removal efficiency, methane production rate and pH [111]. More elaborately, a higher OLR beyond the optimum capacity elevates the rate of production of intermediary products (fatty acids) by hydrolytic and acidogenic bacteria. Subsequently, these fatty acids would accumulate due to the slow rate of their consumption by methanogens thus; pH will drop thereby inhibiting methanogenic activity [112]. Furthermore, Rincòn *et al*. [113] documented that a higher OLR influenced the bacterial community within the digester system; with the genus *Clostridium* being predominant at low OLR and the classes and phyla; *Gammaproteobacteria*, *Deferribacteres*, *Actinobacteria* and *Bacteroidetes* respectively, predominated at high OLR.

#### 3.4.5. Substrate Characteristics and Heavy Metals

The constituents of manure directly determine the biogas yield and the level of biochemical reactions that would take place within the digester system [28]. The composition of the manure will depend on the livestock operations which includes the diet and the handling/storage procedure of the wastes [8]. Evidently, for the proper functioning and continuous reproduction of microbes implicated in the anaerobic digestion process, there is a need for available sources of energy; carbon for the synthesis of new cellular materials, inorganic elements such as nitrogen, phosphorus, potassium, sulfur, calcium and magnesium as well as organic nutrients [114]. As a consequence, the physical and chemical characteristics including the moisture content, total solids content, volatile solids content, phosphorus, nitrogen and carbon content of the feedstock must be evaluated before commencement of the digestion process [40]. 

Volatile solids of manure are a very critical parameter as it consists of the biodegradable portion which includes carbohydrates, fats and proteins and the refractory portion which cannot be anaerobically digested described as lignocellulosic [28]. The term biodegradability of manure is indicated by biogas or methane yield and percentage of solids (total or volatile solids) that are destroyed in the anaerobic digestion process [115]. 

Microorganisms require a trace amount of some metals (nickel, cobalt, copper, iron, zinc, molybdenum *etc*.) for optimum growth and performance. Matseh [116] noted that these trace elements are usually known as stimulatory micro-nutrients and do occur in coenzymes and cofactors. The stimulatory effects potentiated by these metals on biogas process performance are linked to increased methane production, substrate utilization and reactor stability. However, there are wide ranges in the quantity of these metals that are needed in order to become stimulatory; this has been ascribed to differences in pH, OLR, HRT, substrate characteristics and the complex chemical and biological processes monitoring trace metal bioavailability [117]. 

Moreover, the stimulatory effect varies between the different types of trace metals. This is affirmed by the work of Pobeheim *et al*. [118] which recorded an increase in methane yield upon addition of a well-defined trace element solution composed of Co, Ni and Mo. Whereas a higher decrease in methane generation and process stability was noted due to the elimination of Ni from the solution. Results further revealed that 0.4–2 µM concentration of Co caused a 10% increase in methane production but the addition of Mo exhibited no profound effect on methane production.

Interestingly, animal manure has been reported to contain a good level of both macro and micronutrients (trace elements) [23]. However, process failure caused by trace element deficiency has been demonstrated during anaerobic digestion of single substrates such as maize silage. Some mono substrates (e.g., maize silage, potato *etc*.) or even food wastes cannot provide both the micro and macro nutrients essential for the growth of anaerobic microbes that are present in the anaerobic digestion process [119]. Therefore, they have to be supplemented with these nutrients before commencement of the digestion process. Better still, they can be co-digested with animal manure such that animal manure provides good buffering capacity and required nutrients whilst the energy crop provides increases in the energy yield of the process [23,119]. 

A deficiency of these metals can cause shifts in microbial community structure; Gustavsson *et al*. [117] documented a shift in microbial community structure from *Methanosarcinales* dominance during a stable process performance with Co and Ni supplementation to *Methanomicrobiales* dominance at both Co and Ni deficiency. However, too high a concentration of these heavy metals would lead to toxicity of the system thereby hampering the biological process via the enzymes involved by interfering with their function and structure. Apparently, they may substitute for naturally occurring metals in an enzyme prosthetic group or by binding to the SH group of the enzyme [106].

#### 3.4.6. Mixing

Of great value in the anaerobic digestion of animal manure is the extent of contact between the incoming animal manure and a viable bacterial population; this is a function of mixing in the reactor [110]. The benefits of mixing digester contents during anaerobic process have been documented by several authors and include: it prevents scum formation inside the digester, ensures uniform distribution of microorganisms and substrate throughout the mixture and intensifies contact between them, prevents stratification within the digester therefore enables uniform distribution of heat throughout the mixture and lastly helps to release gas from the mixture [30,120].

According to Ghanimeh *et al*. [121], stirring can result in reduction of particle sizes of the substrate as anaerobic digestion progresses. However, what is unclear about the aspect of mixing is the intensity and the duration of mixing considering the fact that different modes (mechanical mixers and recirculation pumps) could be used [110]. In the characterization of manure, total and volatile solids are very paramount because there is a certain limit above which the manure will no longer be a slurry hence posing problems of mixing and pumping operations [28]. As a result, Rico *et al*. [30] mentioned that low total solids added to long HRT minimizes the need of mixing in anaerobic digestion of dairy manure.

## 4. Conclusions

Anaerobic digestion of animal manure is looked upon as a strong option in safely reusing wastes or transforming them into valuable materials and energy. This decomposition process that occurs within a confinement contributes to pollution control as it presents with the following benefits; it reduces biological oxygen demand (BOD) and chemical oxygen demand (COD) of wastes; it destroys pathogenic microbes reducing the microbial load to a level which could be safely handled by humans with limited health risks [16] and it destroys volatile fatty acids and many malodorous compounds present in the feedstock and reduces the emission of greenhouse gases. Ultimately, it generates biogas and high quality nutrient-rich fertilizer from animal manure thus upholds the concept of waste to wealth in enhancing sustainability of development [3].
